# A Survey of Practice and Factors Affecting Physiotherapist-Led Health Promotion for People at Risk or with Cardiovascular Disease in Cameroon

**DOI:** 10.3390/clinpract14050140

**Published:** 2024-08-29

**Authors:** Etienne Ngeh Ngeh, Sionnadh McLean, Christopher Kuaban, Rachel Young, Joanne Lidster

**Affiliations:** 1 Research Organization for Health Education and Rehabilitation-Cameroon (ROHER-CAM), Mankon, Bamenda P.O. Box 818, Cameroon; 2 Department of Allied Health Professions, Sheffield Hallam University, L108, 36 Collegiate Crescent, Sheffield S10 2BP, UK; r.young@shu.ac.uk (R.Y.); j.lidster@shu.ac.uk (J.L.); 3 School of Allied Health Sciences, Charles Darwin University, Darwin, NT 0810, Australia; sionnadh.mclean@cdu.edu.au; 4 Faculty of Medicine and Biomedical Sciences, University of Yaounde I, Yaounde P.O. Box 4021, Cameroon; ckuaban@yahoo.fr

**Keywords:** physiotherapy, health promotion, risk factors, cardiovascular diseases, Cameroon

## Abstract

Background: Cardiovascular diseases (CVDs) and associated risk factors are a growing concern in Cameroon. Physiotherapists (PTs) can play a crucial role in prevention and management. However, the extent of Cameroonian PT involvement in health promotion (HP) activities remains unclear. This study assessed Cameroonian physiotherapists’ current HP practices for people at risk of or with CVDs (pwCVDs). Methods: A cross-sectional survey was administered online to PTs practising in Cameroon. Results: Out of 181 PT responses, 95% reported providing a variety of HP activities, including weight management (74%), dietary advice (73%), physical activity (69%), smoking cessation (69%), stress management (61%), and sleep promotion (48%). While PTs were confident in lifestyle assessments, they felt less confident about sleep interventions. Strong beliefs, confidence, team support, and time allocation enhanced HP practice. However, preference for passive modalities, patient adherence issues, organisational challenges, role ambiguity among healthcare providers, inadequate training opportunities, and the absence of established guidelines for CVD prevention negatively affect HP practice. Conclusions: These findings highlight the challenges and opportunities for enhancing HP delivery within the physiotherapy profession in Cameroon. The findings are useful for future strategies by clinical practitioners and policy makers to address barriers and leverage facilitators effectively for scaling up HP initiatives in Cameroon.

## 1. Introduction

More than two-thirds of all global deaths are attributed to four chronic non-communicable diseases (CNCDs): cardiovascular disease (CVD), cancer, chronic lung diseases, and diabetes [1]. CVD is the leading cause of death globally, with an estimated 20.5 million deaths in 2021 [2], and accounts for 38% of all global premature deaths [1,3]. The burden of CVD is increasing, with over 80% of all global cases and deaths in low- and medium-income countries (LMICs) [3,4]. Although risk factors are similar globally, CVDs are increasing in LMICs due to changing lifestyles (Westernisation) and health behaviours, including physical inactivity, increased use of tobacco, poor nutrition, obesity, and harmful use of alcohol [5]. These, together with other medical risk factors such as hypertension, dyslipidaemia, and diabetes associated with limited resources for effective prevention and management, contribute to the escalating prevalence and mortality of CVDs in LMICs like Cameroon [6].

In Cameroon, CVDs and associated risk factors are on the rise. According to 2015 estimates, 30.8% of Cameroonian women lived with hypertension, compared with 27.0% for both genders in Africa and 20.1% globally [7], accounting for 41.3–54.5% of heart diseases in Cameroon [8,9]. CVD accounted for 10–16% of hospital admissions, with heart failure (38.5%), stroke (33.3%), and uncontrolled hypertension (22.4%) being the most common [9]. In 2012, 12% of total deaths in Cameroon were attributed to CVD [10]. On average, 43.8% of adult Cameroonian males use tobacco, compared to 36.1% globally. Additionally, 28.5% of adults are physically inactive compared to 27.5% globally [11]. A community-based cross-sectional study among adults reported poor awareness of CVDs and associated risk factors among participants [12]. The fragile health system, limited health promotion (HP), disease prevention policies, and implementation might contribute to the increasing prevalence of CVD risk factors [13]. In this work, we defined HP as any proactive measures to improve patients’ quality of life and health.

Most CVD risk factors are modifiable and can be prevented by addressing lifestyle changes such as tobacco use, unhealthy diet and obesity, harmful use of alcohol, poor stress management, physical inactivity, and poor sleep quality [14]. These risk factors can be reduced and prevented through relevant HP practices that enable people to increase control over and improve their health [15,16]. Physiotherapists (PTs) support patients with different conditions, including people at risk of developing or living with CVDs (pwCVDs) [17]. We defined pwCVDs as PT clients with lifestyle risk factors (smokers, alcohol use, and physical inactivity), medical risk factors (diabetes and hypertension), and diagnosed CVD (coronary artery disease and heart failure) [5]. PTs have regular/frequent opportunities to provide their clients with HP advice. As an informed non-medical profession, with health education, physical interventions, and exercise at the core of their practice [18,19], PTs can support management of these lifestyle-related conditions [20,21] using biopsychosocial and holistic approaches [22]. PTs may be an untapped resource for addressing the CVD epidemic in Cameroon, especially with the absence of specialised units and CVD prevention programmes [23]. PT-led health promotion (PLHP) refers to the involvement of PTs in designing, implementing, and guiding strategies and interventions that promote overall health, prevent injuries, and enhance well-being [24]. Personal, professional, institutional, and community policies and clinical guidelines have been shown to influence PT HP practice elsewhere [25,26,27]. The extent to which HP is covered in the curriculum and training of PTs and their HP practice in Cameroon is not known. 

This study aimed to evaluate current PLHP practices and factors affecting PLHP for pwCVDs in Cameroon. The results may help develop and propose strategies and guidelines to improve PLHP practices and health outcomes for pwCVDs in the country.

## 2. Materials and Methods

### 2.1. Study Design

This study was designed as an online cross-sectional survey to assess HP practice by PTs and factors affecting PLHP activities in Cameroon. 

### 2.2. Study Area

The study area included all private and public health facilities in Cameroon where PTs practise. Most PTs are concentrated in urban areas, with very few physiotherapy services delivered in rural areas [17].

Cameroon Society of Physiotherapy (CASP) assumes the governing and regulatory professional role for PTs in Cameroon [28]. CASP does not have complete data for PTs, but previous studies estimate between 250 and 400 PTs are practising in Cameroon [29,30].

### 2.3. Study Population

The study population included PTs who are 21 years and above, practising in Cameroon in any region, including public, private, or mission health facilities, and have at least two years of physiotherapy training and one year of clinical practice. Eligible participants had to be able to read and understand English.

### 2.4. Sample Size

A non-probability (convenience) sampling method was used for this survey due to challenges in determining the number of potential participants. CASP data on the number and characteristics of PTs in Cameroon is incomplete, with only 44 PTs having complete data. The existing literature estimates the number of PTs in Cameroon to vary between 250 and 400 [29,30]. Consequently, the minimum sample size estimated for this study was based on the sample size of 141 study participants used in a similar study in Nigeria [31]. 

### 2.5. Survey Instrument 

The survey development was informed by previous studies [27,32] and guidelines in survey development [33]. The survey was designed to capture data in three main areas: Section A: demographics; Section B: current health promotion practice; and Section C: factors affecting HP practice at four ecological levels [27]. Demographic variables included age, gender, training, duration of training and clinical experience, health sector, and region of practice. Data on current practice included exercise and physical activity promotion, dietary advice, weight management, smoking cessation, sleep, stress management, and level of confidence in promoting them. In Section C, a four-level ecological model was used to assess factors affecting HP practice, and data on intrapersonal, interpersonal, institutional, and community influences were collected. Closed questions were used to enable management and analysis of responses following guidelines for development and reporting of the survey [33,34]. The developed survey instrument was pilot-tested on a sample of 5 PTs practising in Cameroon who were not included in the study. All feedback was integrated to improve the survey instrument.

### 2.6. Recruitment and Data Collection

The recruitment of the study participants and data collection was conducted from 1 November 2023 to 31 January 2024. For recruitment, we used the CASP register or mailing list to identify eligible PTs for the study. This group of PTs was asked to promote the survey through their physiotherapy social media networks and community of practice groups at regional and national levels to facilitate recruitment of other PTs practising in Cameroon but who have yet to register with CASP (snowballing) [33]. The survey instrument was administered online using Qualtrics. Participants were able to complete the survey online using electronic devices such as laptops, iPads, and cell phones [33]. Reminders were sent every two weeks during the period the survey was live to ensure maximum response.

### 2.7. Data Analysis

The web-based data collected were screened for complete responses and downloaded in a Microsoft Excel 2021 document format by the lead author. The data were then transferred to SPSS (IBM SPSS Statistical Software, version 23.0) for statistical analysis. Descriptive statistics (frequencies, central tendency, dispersion/variation, and percentages) were used to present the demographic information and pattern of practice among participants. All missing data (either due to omitted responses or the ‘not applicable’ option) were considered in the analysis.

## 3. Results

### 3.1. Participant Characteristics

Responses to the survey questionnaire were obtained from a total of 230 participants. Of these, 38 (16.5.%) were incomplete or poorly completed, and 11 (4.7%) respondents did not meet the inclusion criteria and therefore were not included in the analysis. Table 1 presents the general characteristics of the study participants. Of the 181 respondents included in the analysis, 53% (*n* = 96) were male and 47% (*n* = 85) female, with a mean age of 34.43 ± 9.0 years and a mean working experience of 8.96 ± 6.0 years. The mean number of PTs per service was 4.31 ± 4.60, with the highest responses coming from the central region of Cameroon (37.6%, *n* = 68). Most respondents were trained in Cameroon (95.6%, *n* = 173) and based in urban settlements (86.7%, *n* = 157). Of the total sample, 19.3% (*n* = 35) had a clinical speciality, with the highest being sport (*n* = 11). Most respondents practised in the private sector, 40.3% (*n* = 73), with 28.2% working in both the public and private sectors. 

### 3.2. PT Practice and Levels of Confidence of HP for PwCVDs

PT HP practice is reported in Table 2. Overall, 95% (*n* = 172) of participants reported that they provide HP to pwCVDs. Most respondents reported that they always conduct HP around weight management (74%, *n* = 123), dietary advice to increase fruit intake (73%, *n* = 127), and exercise and physical activity (69%, *n* = 118). Fewer PTs deliver education around BMI (58%, *n* = 98) and sleep (48%, *n* = 83).

**Figure 1:**
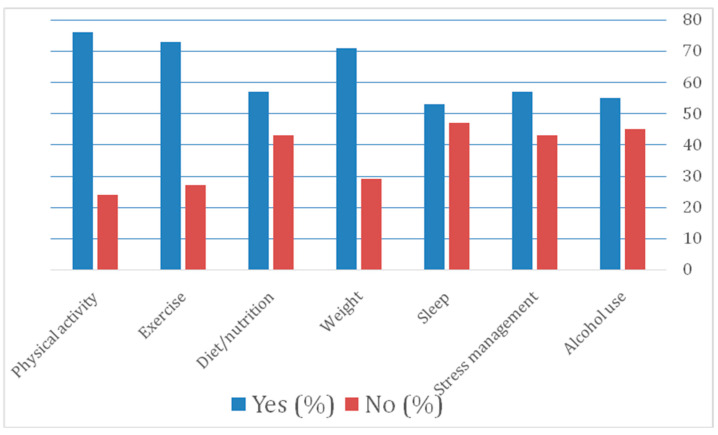
Physiotherapists’ awareness of public and clinical guidelines (*n* = 172).

**Figure 2:**
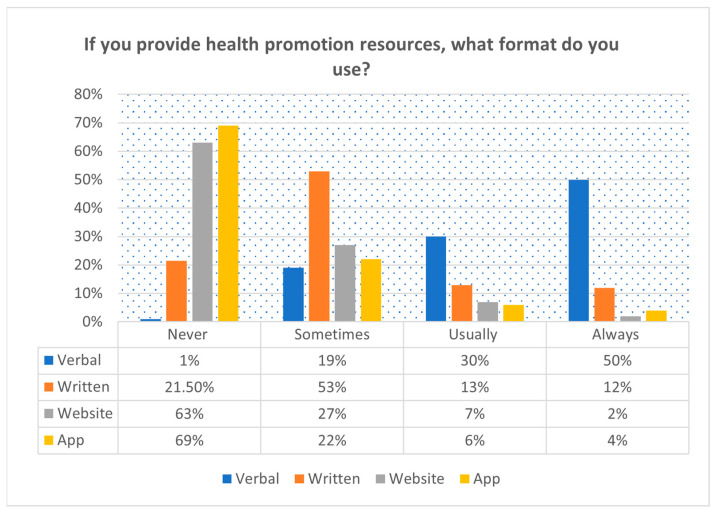
Format of health promotion practice for people at risk of or with cardiovascular diseases of 172 physiotherapists in Cameroon.

The majority of respondents were aware of public and clinical guidelines for physical activity (76%, *n* = 130), exercise (73%, *n* = 124), and weight management (71%, *n* = 121), compared to sleep at 53% (*n* = 91) (see Figure 1). Most PTs deliver HP advice verbally (80%), with apps (69%, *n* = 118) and websites (63%, *n* = 109) never used (Figure 2).

Table 3 provides details on PTs’ confidence level, with the majority confident in assessing lifestyle elements such as weight management (64%, *n* = 110) and alcohol use (56%, *n* = 95). PTs reported being slightly confident or lacking confidence in undertaking HP activities involving sleep (61%, *n* = 102) and diet (50%, *n* = 85). Overall, 45–69% of the respondents indicated that they assess the level of confidence of pwCVDs to engage in HP in their practice, with 9–20% reporting that they have never assessed the confidence level to improve lifestyle changes for pwCVDs. In addition, 46–61% of the respondents reported that they always address challenges pwCVDs may face across several components of HP and provide them with optimal recommended values in areas such as physical activity, details in Supplementary Table S1.

### 3.3. Factors Affecting HP Practice among PTs in Cameroon for pwCVDs

Factors affecting HP are detailed in Table 4. At the intrapersonal level, professional beliefs about using HP only for those at high risk were held by 64% of respondents (*n* = 110) and using passive modalities in practice by 30% (*n* = 53), which may negatively affect HP practice. Participants reported having the relevant skills and knowledge to deliver HP (68%, *n* = 123) and believe that disease prevention and lifestyle modification are essential for managing chronic conditions (63%, *n* = 110). At the interpersonal level, the use of personal lifestyle experiences for HP reported by 76% of respondents (*n* = 131), lack of skills and economic resources (28%, *n* = 49), and pwCVDs not adhering to HP recommendations (25%, *n* = 42) may negatively affect HP practice. Interpersonal factors include having confidence in team members, reported by 70% (*n* = 119), and aligning HP interventions with reasons for consultations, reported by 75% (*n* = 128) of the respondents. Factors hindering HP practice at the institutional level include lack of organised practice (53%, *n* = 90), poor role clarification (32%, *n* = 55), and the absence of regular training opportunities on HP (40%, *n* = 68). The primary institutional factors include favourable working conditions (52%, *n* = 89) and having time for HP practice (59%, *n* = 100) even without financial incentives (59%, *n* = 100). The lack of existing guidelines on CVD prevention (28%, *n* = 49) in the Cameroonian setting may hinder HP practice at the community and public levels. Non-interference of religion (61%, *n* = 104) and culture (59%, *n* = 102) on HP among respondents may enhance practice. 

## 4. Discussion

To our knowledge, this is the first study to evaluate practice and factors affecting PLHP for pwCVDs in Cameroon. PTs reported generally high levels of HP practice and confidence to deliver on several components.

### 4.1. PT Practice of HP for PwCVDs by PTs

The majority of PTs in Cameroon report that they currently deliver several HP components for pwCVDs. Higher proportions of PTs reported delivering on weight management (74%) and dietary advice to eat more fruit (73%) than exercise and physical activities (69%) and sleep (48%). Previous African studies have focused on knowledge, attitudes, and practices towards HP and physical activities among PTs [35]. Studies conducted in Ghana and Nigeria reported that 87% and 92.2% of PTs, respectively, incorporate some aspect of HP in the delivery of care [36,37]. While few studies focused on specific components of HP, some studies have reported similar percentages of integrating HP in practice. Many reported values of HPs were higher than those reported in some African and high-income countries [35,38,39]. For instance, studies conducted in Ghana and Rwanda reported similar values regarding diet and physical activity recommendations [37,38]. Our respondents also reported a higher integration of HP practice in physical activities, smoking cessation, and sleep than in studies conducted in Nigeria, Canada, and Jordan [39,40,41]. Despite 81.5% agreeing that nutritional/dietary counselling is within their scope of practice, less than 50% of Irish and Australian PTs assessed the nutritional status of patients, and even fewer PTs provided nutritional interventions [42,43,44]. The generally lower reported HP activities in previous studies may be due to being conducted in the general patient populations rather than pwCVDs. In pwCVDs, PTs may be more likely to consider the risk and lifestyle factors and what they can do in their role as PTs. It is therefore necessary that studies evaluating the practice of HP for individual components, such as nutritional/dietary counselling, be conducted among PTs in Cameroon to obtain a clearer picture.

### 4.2. PT Levels of Confidence to Deliver HP for PwCVDs

Consistent with prior research, our data indicate lower confidence levels in delivering HP interventions across all reported behaviours [39,40,41]. We report a similar level of confidence (50%) in delivering dietary advice as in Nigeria (50%) [45] and lower levels of confidence in delivering physical activity (53%) compared to 75% and 93% reported in Nigeria and Australia, respectively [39,43]. The lower level of confidence in physical activity is concerning as this is a primary focus in PT practice. A lack of physical activity policies and implementation in Cameroon may explain this [13]. We reported higher levels of confidence in delivering advice on alcohol use (56%) compared to Nigeria (42.5%) [45] and sleep (39%) compared to reports from Jordan (12%) [41,46]. Conversely, 57% of United States PTs reported routinely assessing their patient’s sleep behaviours [46]. The difference in confidence levels reported across HP components could be accounted for by the different study populations, pwCVDs in our case, and patients in general physiotherapy practice in most previous studies. Also, the purpose and design of data collection instruments in those studies may influence reported outcomes even in a similar context like Nigeria and Cameroon. The focus of our study on pwCVDs might have influenced PTs to think and report their role in modifiable risk factors, not their actual confidence in practice, leading to respondent bias in the study. The PTs in the United States might have access to more formal training, clinical guidelines, and collaborating experts (dieticians/nutritionists, clinical sleep specialists, and psychologists) providing support and increasing confidence in their role in HP practice [46].

Our data align with findings among healthcare students in England; confidence in delivering HP interventions increases in areas of practice with specialists and transparent referral processes [47]. Also, the availability and volume of training in undergraduate physiotherapy courses to deal with a range of HP activities is likely to impact PTs’ confidence in delivering HP activities in practice [47].

Physiotherapy practice is usually centred on physical activity and exercise, and with the bio-psychological model, PTs’ scope of practice has been expanding [19]. Areas such as alcohol use, smoking cessation, stress management, and sleep are still being embraced within the scope of PT practice in Cameroon; confidence and skills to deliver in these areas have been historically low in both high- and low-resource settings [41,46,48]. Confidence is associated with training, but the extent to which HP is integrated into physiotherapy training in Cameroon is not known. Lack of relevant training, including guided counselling and behaviour change techniques such as motivational interviewing, may contribute to low levels of confidence, given behaviour change is required across these lifestyle components [49]. Further studies are warranted to assess factors associated with practices and interventions around specific lifestyle factors in Cameroon. Recent studies demonstrate that PT knowledge and experience are limited in different lifestyle behaviours and conditions, including but not limited to smoking, nutrition, sleep, and stress management, with an increasing need for further education/training to address these behaviours [50,51].

PTs predominantly provide verbal advice to pwCVDs, with relatively little written or printed materials, websites, and apps. This may be associated with the need for more resources, proper educational materials, and expertise on HP, making standard reference material challenging. Even the elderly pwCVDs associate with some form of digital device or that of their carers. Recent trials elsewhere demonstrate the high acceptability of technological-based interventions in rural older adults with obesity [52,53]. Despite contextual barriers in Cameroon, technology-based intervention for HP remains a viable option.

### 4.3. Factors Affecting HP Practice for pwCVDs

Consistent with the existing literature, respondents in this study reported that intrapersonal factors such as solid professional beliefs, appropriate skill set and knowledge, motivation, positive attitude, and self-confidence towards HP could potentially improve HP practice (Table 4) [37,42,47]. This favours long-term engagement and practice of HP for pwCVDs in Cameroon and influences how HP issues are perceived and addressed. PTs with strong professional beliefs and skills concerning HP are likely to promote their role at different ecological levels (interpersonal, institutional, and public/community). Our findings align well with the global calls to enhance PTs’ contributions to promoting health in daily practice with relevant competencies across different components of HP [19].

Factors potentially limiting HP practice consistent with previous research were identified [35]. These include the lack of regular training, limited resources, lack of specialist referral pathways, and poor role clarification with other clinicians, similar to reports from other Africa-based studies and elsewhere [35,43,44]. Despite time being commonly cited as a barrier in studies from Africa and high-income countries [35,41], the majority of PTs felt that they had the time and working conditions that would allow them to deliver HP activities [35]; this may explain the relatively high level of reported HP activities. This suggests that the priority given to the lack of knowledge, skills, resources, and confidence in improving specific behaviours outweighs the importance of practice time, underscoring the severity of these factors. The situation may vary in other countries that operate under more stringently managed systems. The limited influence of time on HP is encouraging, but it also reveals other challenges related to diverse sectors, healthcare policies, and future practices. For instance, in the private sector, where patients pay for each visit, they may resist follow-up visits due to financial concerns. On the other hand, in the public sector, where services are often free or subsidised, follow-up visits may be constrained by workload and limited contact time per visit [26]. 

### 4.4. Implications for Practice

#### 4.4.1. Clinical

The current HP practice and the PTs’ confidence level in Cameroon highlight the need for evidence-based training to design effective interventions and support behaviour change. Advice alone is insufficient for sustained behaviour change, indicating a shift towards health coaching practices [16]. Effective HP acknowledges patients as experts in their situation, with PTs acting as coaches to instil purpose and confidence [54].

This study demonstrates the complex skill set that is required by PTs to address the multifactorial needs of pwCVDs by identifying different areas of awareness and factors affecting HP practice. These are necessary to provide patient-centred approaches and personalised interventions tailored to individual needs. PTs must appreciate the nuanced ways in which these risk factors affect patients with CVDs, necessitating personalised interventions for optimal outcomes. In line with the global call for PTs to address the increasing global burden of CNCDs [19,20], PTs are challenged to address several risk factors. The World Health Professions Alliance provides resources such as the Health Improvement Card, which can be used to assess and monitor risk factors for pwCVDs [55].

#### 4.4.2. Educational

The findings reveal that PTs conduct HP activities but lack confidence across multiple domains. Cameroonian PTs may need more knowledge or confidence in utilising behaviour change techniques to address lifestyle conditions for pwCVDs. This highlights the need for formal training at entry level into practice and continuous professional development training on HP and behaviour change techniques to effectively equip PTs with the requisite skills and knowledge to address lifestyle-related issues [43,48]. In line with existing evidence and ecological models, efforts should focus on strengthening individual factors and professional beliefs among PTs to positively impact healthcare [21].

#### 4.4.3. Policy

The findings offer valuable insights for the development and implementation of PLHP initiatives not only in Cameroon but also in similar settings in Africa. They underscore the importance of curriculum development that integrates HP strategies to equip PTs to address the CVD pandemic. Entry-level physiotherapy programmes in Cameroon should be assessed to ensure they equip graduates with the skills to address emerging healthcare needs, such as promoting health and preventing illnesses, especially among pwCVDs. The government should invest in producing evidence-based public and clinical guidelines in Cameroon.

### 4.5. Strengths and Limitations

Strengths include the use of a rigorously developed and validated survey instrument, the use of ecological levels to investigate factors affecting HP practice holistically, the large number of respondents, and the use of recommended guidelines in the conduct and reporting of this study.

The study faced challenges with the representativeness of its sample, which makes generalising the findings difficult. This was mitigated by recruiting a large sample of practising PTs, estimated at 250–400 [29,30]. Secondly, respondent bias is possible, as individuals who completed the survey may have a heightened interest in HP activities [56]. This bias could skew the findings and may not accurately represent the broader population of PTs in Cameroon. Thirdly, the sample was not specific to PTs who manage pwCVDs. PTs with much lower caseloads of pwCVDs may face different realities in their practice, so findings should be considered cautiously. Fourthly, we examine potential factors affecting several components of HP without any solid association with PLHP practice; future studies should examine barriers and facilitators to specific components of PLHP in Cameroon. Finally, while the study utilised a validated survey tool, the reliance on quantitative research methods with closed-ended questions may limit the depth of insights into PTs’ beliefs, attitudes, and perceptions regarding HP for pwCVDs. This limitation underscores the need for complementary qualitative research to explore these aspects comprehensively.

## 5. Conclusions

Cameroonian PTs reported high levels of practice in some HP activities with consistently lower confidence levels in delivering all activities for pwCVDs. Most respondents delivered HP advice verbally, seldom using print or written advice. Professional beliefs, confidence in interventions, supportive teams, and favourable working conditions potentially enhance HP practice. This highlights the necessity of strong organisational and professional support. However, entrenched beliefs in using passive modalities, patient adherence issues, and systemic challenges, including lack of resources, guidelines, role ambiguity, and limited training opportunities, may limit HP practice. 

This study demonstrates the complex skill set that PTs require to effectively address the multifactorial needs of pwCVDs by identifying different areas, awareness, and factors affecting HP practice. In line with existing evidence and ecological models, efforts should focus on strengthening individual factors and professional beliefs among PTs to positively impact the broader healthcare ecosystem. This highlights the importance of curriculum development integrating HP strategies to equip PTs to address the CVD pandemic. Our quantitative research methods with closed-ended questions may limit the depth of insights into Cameroonian PTs’ beliefs, attitudes, and perceptions regarding HP for pwCVDs. This limitation underscores the need for complementary qualitative research to gain an in-depth understanding of HP practices in Cameroon.

## Figures and Tables

**Table 1:** Socio-demographic characteristics of the 181 physiotherapists enrolled in the study.

Variable		*n*	%
Sex	Male	96	53.0
	Female	85	47.0
Mean age (years)	34.43 ± 9.0	181	100
Mean working duration	8.96 ±6.0	181	100
Mean number of PTs per service	4.31 ± 4.60	181	100
Educational level	Higher Diploma	75	41.4
	Bachelor’s Degree	81	44.4
	Master’s Degree	22	12.2
	Others	3	1.7
Location of participants	Central	68	37.6
	East	4	2.2
	Far North	3	1.7
	Littoral	51	28.2
	North	1	0.6
	North-West	27	14.9
	South-West	21	11.6
	West	6	3.3
Location of training	Cameroon	173	95.6
	Africa	4	2.3
	Europe	3	1.7
	United States	1	0.6
Clinical specialism	Musculoskeletal	7	3.9
	Cardiorespiratory	3	1.7
	Neurology	6	3.3
	Paediatric	3	1.7
	Sports	11	6.1
	No speciality	151	83.4
Settlement	Rural	24	13.3
	Urban	157	86.7
Sector of work	Private	73	40.3
	Public	51	28.2
	Both private and public	51	28.2
	Others (NGOs and missionary hospitals)	6	3.3
Current institution of practice	Tertiary hospital	29	16.0
	Secondary/regional hospital	29	16.0
	District hospital	23	12.7
	Community health centre	4	2.2
	Clinic	54	29.8
	Rehabilitation centre	32	17.7
	Special school	2	1.1
	Education/university	13	7.2
	Others	19	10.5

Notes: PT; physiotherapist, NGO; non-governmental organisation.

**Table 2:** Health promotion practices of 172 physiotherapists for people at risk of or with cardiovascular disease in Cameroon.

Please Indicate Which of the Relevant Areas Best Describes Your Interventions.			
	Never	Sometimes	Always
Discuss weight management	8% (*n* = 14)	18% (*n* = 30)	74% (*n* = 123)
Dietary advice regarding eating more fruits	6% (*n* = 11)	21% (*n* = 35)	73% (*n* = 127)
Increase general physical activity	2% (*n* = 3)	30% (*n* = 51)	69% (*n* = 118)
Increase specific exercise uptake	3% (*n* = 5)	28% (*n* = 48)	69% (*n* = 118)
Encourage the patient to stop smoking	11% (*n* = 18)	21% (*n* = 35)	69% (*n* = 116)
Dietary advice to decrease excessive salt use	13% (*n* = 22)	22% (*n* = 38)	65% (*n* = 111)
Condition-specific education for patients with Known chronic Cardiovascular Conditions	8% (*n* = 14)	28% (*n* = 47)	64% (*n* = 109)
Counsel to manage stress	9% (*n* = 16)	30% (*n* = 50)	61% (*n* = 103)
Dietary advice regarding eating more vegetables	9% (*n* = 15)	32% (*n* = 54)	60% (*n* = 102)
Explain the value of BMI as a measure of health	13% (*n* = 22)	29% (*n* = 49)	58% (*n* = 98)
Education around normal sleeping patterns	17% (*n* = 29)	34% (*n* = 57)	48% (*n* = 83)

**Table 3:** Physiotherapists’ level of confidence engaging in health promotion practice.

Are You Confident in Assessing the Lifestyle and Behaviour of People at Risk or with CVDs?			
	Not Confident at All	Slightly Confident	Confident
Weight	14% (*n* = 24)	22% (*n* = 38)	64% (*n* = 110)
Alcohol use	19% (*n* = 33)	25% (*n* = 43)	56% (*n* = 95)
Physical activity	11% (*n* = 18)	36% (*n* = 61)	53% (*n* = 92)
Stress management	19% (*n* = 32)	29% (*n* = 50)	52% (*n* = 95)
Diet	15% (*n* = 26)	35% (*n* = 59)	50% (*n* = 84)
Sleep	24% (*n* = 41)	36% (*n* = 61)	39% (*n* = 67)

**Table 4:** Factors affecting HP practice based on respondents (*n* = 172).

To What Extent Do You Agree with the Following Statements:			
**POTENTIALLY LIMITING HP PRACTICE**			
**Intrapersonal Factors**	**Disagree**	**Undecided**	**Agree**
I commonly use health promotion for those at high risk of CVD or those with complications only	15% (*n* = 26)	21% (*n* = 36)	64% (*n* = 110)
I believe the professional role of physiotherapist is to primarily treat patients using passive modalities	42% (*n* = 72)	27% (*n* = 47)	30% (*n* = 53)
**Interpersonal factors**			
I lack the skills and economic resources to implement health promotion in my practice	51% (*n* = 87)	21% (*n* = 36)	28% (*n* = 49)
I use my personal lifestyle experiences to facilitate health promotion for patients	6% (*n* = 11)	17% (*n* = 30)	76% (*n* = 131)
My patients lack the interest to adhere to health promotion recommendations	40% (*n* = 68)	36% (*n* = 61)	25% (*n* = 42)
Institutional factors			
I have regular training (continuous professional development) in health promotion	35% (*n* = 60)	25% (*n* = 43)	40% (*n* = 68)
There are no resources on health promotion in my institution	53% (*n* = 90)	24% (*n* = 40)	23% (*n* = 39)
We lack an organised practice with referral units like nutrition service and counselling units	27% (*n* = 47)	20% (*n* = 35)	53% (*n* = 90)
The lack of role clarification with other healthcare providers hinders health promotion practice in my institution	41% (*n* = 70)	27% (*n* = 46)	32% (*n* = 55)
I lack office space for health promotion	51% (*n* = 88)	22% (*n* = 38)	27% (*n* = 46)
Community/public factors			
There are no existing guidelines for prevention of CVD in our setting	48% (*n* = 82)	24% (*n* = 41)	28% (*n* = 49)
There are no physiotherapy health promotion practices in public hospitals or settings	48% (*n* = 82)	30% (*n* = 52)	22% (*n* = 38)
There are no physiotherapy health promotion practices in a private hospital	42% (*n* = 73)	40% (*n* = 69)	17% (*n* = 30)
**POTENTIALLY ENHANCING HP PRACTICE**			
**Intrapersonal Factors**	**Disagree**	**Undecided**	**Agree**
Medical management is more important than lifestyle modification for chronic conditions	63% (*n* = 110)	22% (*n* = 37)	15% (*n* = 25)
I am confident I have appropriate skills and knowledge on health promotion and disease prevention	6% (*n* = 11)	22% (*n* = 38)	68% (*n* = 123)
Because of my personal difficulty dealing with a lifestyle issue like being overweight, smoking, etc, I find it difficult to talk about similar issues with my patients	67% (*n* = 115)	19% (*n* = 32)	14% (*n* = 24)
I normally do not waste my time on health promotion for patients as it will not be effective	74% (*n* = 127)	12% (*n* = 21)	13% (*n* = 22)
I believe that there will be no change in patients’ behaviour even if I provide lifestyle recommendations	71% (*n* = 121)	13% (*n* = 23)	16% (*n* = 28)
**Interpersonal factors**			
I have confidence in my team members and colleagues to assist me in implementing health promotion in my practice	8% (*n* = 14)	22% (*n* = 38)	70% (*n* = 119)
I do not Practise health promotion because it will conflict with the original reason for patient consultation	75% (*n* = 128)	13% (*n* = 22)	12% (*n* = 21)
Institutional factors			
I lack the time to implement health promotion in my practice	59% (*n* = 100)	24% (*n* = 40)	17% (*n* = 30)
My working conditions do not permit me to implement health promotion	52% (*n* = 89)	28(*n* = 48)	20% (*n* = 34)
I do not practice health promotion because there are no financial benefits to health promotion	66% (*n* = 113)	18% (*n* = 31)	16% (*n* = 28)
Community/public factors			
Because of the social class or status of some patients, I find it difficult to discuss health promotion recommendations	56% (*n* = 97)	25% (*n* = 43)	19% (*n* = 32)
Religious practices make it difficult for me to promote health in my practice	61% (*n* = 104)	20% (*n* = 35)	19% (*n* = 33)
Because of cultural practices and language, I find it challenging to implement health promotion in my practice	59% (*n* = 102)	23% (*n* = 39)	18% (*n* = 31)

## Data Availability

The datasets generated during and analysed during the current study are available from the corresponding author upon reasonable request.

## Supplementary Materials

The following supporting information can be downloaded at: https://www.mdpi.com/article/10.3390/clinpract14050140/s1, Table S1: A Do you ask your patients about their confidence to change or improve their habits in the following areas? B Do you discuss challenges patients may face while trying to improve in any of the following areas? C Do you assist your patient to know the optimal recommended values for? What is most (highest) common type of problems your see on daily basis?—Selected Choice.
