# Supplementary material for: A Survey of Practice and Factors Affecting Physiotherapist-Led Health Promotion for People at Risk or with Cardiovascular Disease in Cameroon
Source: Clin Pract. 2024 Aug 29;14(5):1753–66. doi: 10.3390/clinpract14050140 (PMC11417807; doi:10.3390/clinpract14050140)
Supplement: Supplementary file 1 [file clinpract-14-00140-s001.zip › clinpract-3116027-supplementary.pdf]

Supplementary file Table S1

A

Do you ask your patients about their confidence to change or improve their habits in the following areas?					
	Never	Sometimes	Usually,	Always	
Alcohol intake	17%(n=29)	27%(n=45)	26%(n=44)	30%(n=51)	
Physical activity	9%(n=16)	22%(n=38)	32%(n=54)	37%(n=63)	
Diet	14%(n=24)	23%(n=39)	36%(n=62)	27%(n=47)	
Weight	16%(n=27)	23%(n=40)	28%(n=49)	33%(n=56)	
Sleep	20%(n=34)	35%(n=61)	26%(n=44)	19%(n=33)	
Stress management	16%(n=27)	32%(n=54)	22%(n=37)	31%(n=52)	

B

Do you discuss challenges patients may face while trying to improve in any of the following areas?				
	Never	Sometimes	Usually	Always
Physical activity	7%(n=12)	33%(n=56)	28%(n=48)	33%(n=56)
Diet	11%(n=19)	31%(n=51)	32%(n=53)	26%(n=44)
Weight	13% (n=22)	28%(n=48)	26%(n=44)	33%(n=56)
Sleep	24%(n=40)	31%(n=52)	26%(n=44)	20%(n=34)
Stress management	18%(n=31)	30%(n=51)	21%(n=36)	30%(n=51)
Alcohol	21%(n=35)	17%(n=45)	24%(n=41)	28%(n=48)

C

Do you assist your patient to know the optimal recommended values for?				
	Never	Sometimes	Usually	Always
Physical activity	11%(n=18)	46%(n=79)	23% (n=40)	20%(n=34)
Diet/nutrition	18%(n=31)	42%(n=72)	23%(n=39)	17%(n=29)

Weight	15%(n=25)	34%(n=58)	28%(n=47)	24%(n=4)
Sleep	22%(n=37)	42%(n=70)	23%(n=39)	13%(n=22)
Stress management	22%(n=38)	36%(n=61)	21%(n=36)	21%(n=35)
Alcohol intake	19%(n=32)	35%(n=59)	23%(n=39)	23%(n=39)

What is most (highest) common type of problems your see on daily basis? - Selected Choice

		Frequency	Percent	Valid Percent	Cumulative Percent
Valid	Orthopedic	70	38.7	38.7	38.7
	Neurological	76	42.0	42.0	80.7
	Cardiovascular and respiratory	1	.6	.6	81.2
	Pediatric (< 15 years)	4	2.2	2.2	83.4
	Geriatric (>65 years)	11	6.1	6.1	89.5
	Sport	5	2.8	2.8	92.3
	Others please, specify	14	7.7	7.7	100.0
	Total	181	100.0	100.0	
